# Systematicity and a Categorical Theory of Cognitive Architecture: Universal Construction in Context

**DOI:** 10.3389/fpsyg.2016.01139

**Published:** 2016-07-29

**Authors:** Steven Phillips, William H. Wilson

**Affiliations:** ^1^Mathematical Neuroinformatics Group, Human Informatics Research Institute, National Institute of Advanced Industrial Science and TechnologyTsukuba, Japan; ^2^School of Computer Science and Engineering, University of New South WalesSydney, NSW, Australia

**Keywords:** systematicity, compositionality, classicism, connectionism, category theory, universal construction, (co)recursion, (co)algebra

## Abstract

Why does the capacity to think certain thoughts imply the capacity to think certain other, structurally related, thoughts? Despite decades of intensive debate, cognitive scientists have yet to reach a consensus on an explanation for this property of cognitive architecture—the basic processes and modes of composition that together afford cognitive capacity—called systematicity. Systematicity is generally considered to involve a capacity to represent/process common structural relations among the equivalently cognizable entities. However, the predominant theoretical approaches to the systematicity problem, i.e., classical (symbolic) and connectionist (subsymbolic), require arbitrary (*ad hoc*) assumptions to derive systematicity. That is, their core principles and assumptions do not provide the necessary and sufficient conditions from which systematicity follows, as required of a causal theory. Hence, these approaches fail to fully explain why systematicity is a (near) universal property of human cognition, albeit in restricted contexts. We review an alternative, category theory approach to the systematicity problem. As a mathematical theory of structure, category theory provides necessary and sufficient conditions for systematicity in the form of universal construction: each systematically related cognitive capacity is composed of a common component and a unique component. Moreover, every universal construction can be viewed as the optimal construction in the given context (category). From this view, universal constructions are derived from learning, as an optimization. The ultimate challenge, then, is to explain the determination of context. If context is a category, then a natural extension toward addressing this question is higher-order category theory, where categories themselves are the objects of construction.

## 1. Introduction: The systematicity challenge

Systematicity is a property of cognitive architecture—the organization of basic processes affording cognition—where the capacity for certain cognitive abilities implies having the capacity for certain related cognitive abilities (Fodor and Pylyshyn, 1988). An often used example of systematicity is where having the capacity to understand the statement *John loves Mary* implies having the capacity to understand the statement *Mary loves John*. This property need not be restricted to language, nor humans. In general, systematicity has been characterized as having cognitive capacity *c*_1_ if and only if one has structurally related cognitive capacity *c*_2_ (McLaughlin, 2009). That is, systematically related cognitive capacities form an equivalence class with respect to certain structural equivalence relations. Despite decades of intensive debate, cognitive scientists have yet to reach a consensus on an explanation for systematicity, as evidenced by the diversity of views in a recent reassessment of the systematicity challenge (Calvo and Symons, 2014).

The challenge for theories of cognitive architecture is to explain why the distributions of cognitive capacities tend to be centered around structural equivalences. That is, to provide necessary and sufficient conditions from which systematicity follows without relying on arbitrary (*ad hoc*) assumptions (Fodor and Pylyshyn, 1988; Aizawa, 2003). In a nutshell, *ad hoc* assumptions are auxiliary assumptions that are motivated solely by the need to fit the data, unconnected to the core theory, and not confirmed independently of confirming the theory (Aizawa, 2003).

Classical (symbol systems) theory was thought to explain systematicity by virtue of a suitable collection of grammatical structures (symbol transformation rules) that afford the target collection of capacities: capacity for one instance implies capacity for all instances, because they are all obtained from one and the same grammar, assuming basic processes for recognizing/generating atomic symbols. Ironically, the classical explanation appears to suffer the same kind of problem that was raised against connectionism: i.e., the *ad hoc* way in which grammatical (cf. neural network) structures can be configured with and without support for systematicity (Aizawa, 2003). One can straightforwardly provide a grammar that supports some but not all the requisite capacities (Phillips and Wilson, 2010). Classicists claim that the only classical grammatical structures are the “canonical grammars” (McLaughlin, 2009). However, what characterizes canonicity, beyond just being the grammars that support systematicity, and what determines having just such canonical grammars is unclear.

The challenge facing the classical approach to theories of cognition echoes the one facing the connectionist and other approaches: develop a theory of cognitive architecture whose core principles and assumptions provide the necessary and sufficient conditions from which systematicity follows, as required of a causal theory (Fodor and Pylyshyn, 1988; Fodor and McLaughlin, 1990; Aizawa, 2003). A common recourse with regard to connectionist approaches is to augment models with a capacity to learn to be systematic as a form of generalization (see e.g., Hadley, 1994; Niklasson and van Gelder, 1994; Hadley and Hayward, 1997; Boden and Niklasson, 2000). For instance, the capacity to understand sentences with constituents in novel syntactic/semantic positions is called *strong systematicity* (Hadley, 1994): e.g., the capacity to infer that Mary is the person being loved in the sentence “John loves Mary” having been trained to make such inferences with Mary only ever appearing in the lover position. However, there are many possible ways to train a neural network, and not all of them support the requisite level of generalization (see e.g., Marcus, 1998; Phillips, 1998). Thus, a challenge for the learning approach is to explain why a network is configured in just the right way to afford the desired generalization property, which echoes the original systematicity problem (Phillips and Wilson, 2010).

If systematicity depends on some kind of structure, then a prospective approach is a mathematical “theory of structure,” in a technical sense to be elaborated upon later, called *category theory* (Eilenberg and Mac Lane, 1945; Mac Lane, 1998). Category theory was originally invented as a formally precise language to compare mathematical objects. However, subsequent developments have transformed category theory into a branch of mathematics in its own right (see Marquis, 2009, for a historical perspective). The main areas of application outside of mathematics have been computer science (see e.g., Arbib and Manes, 1975; Rydeheard and Burstall, 1988; Barr and Wells, 1990; Walters, 1991; Crole, 1993; Bird and de Moor, 1997) and physics (see e.g., Coecke, 2006; Baez and Stay, 2011). Though applications to cognitive science have been relatively few, they include cognitive development (Halford and Wilson, 1980; Phillips et al., 2009), concepts (Magnan and Reyes, 1995; Healy and Caudell, 2004), language (Lambek, 2004; Ellerman, 2007; Clark et al., 2008), memory (Ehresmann and Vanbremeersch, 2007), navigation (Gomez-Ramirez, 2014), and neural models (Healy et al., 2009). We have been advocating a category theory-based explanation for various aspects of systematicity (Phillips and Wilson, 2010, 2011, 2012), see also Halford et al. (2014), ch. 2 and Phillips and Wilson (2014) for an overview. The purpose of the current paper is to review this explanation in so far as it accounts for systematicity and assess what further challenges lay ahead.

Category theory provides an explanation for systematicity in terms of universal constructions (Section 2). This explanation leads one to inquire as to why universal constructions naturally arise. A universal construction can be viewed as an optimal construction in the given context (category). So, we look at the relationship between optimization and learning as the basis for explaining the origins of universal constructions (Section 3). This approach also leads naturally to an algorithm for learning universal constructions that is derived from this category theory perspective (Section 4). Finally, we look at the implications of this (unique) category theory perspective on systematicity, and a new challenge that follows for cognitive science (Section 5).

## 2. A category theory explanation: Universal constructions

Conventional wisdom suggests starting with familiar examples when introducing a new concept. So, one might introduce the concept of a category to a graph theorist as a generalized graph whose nodes and edges may involve additional information beyond labels, to an order theorist as a collection of generalized (partially) ordered elements, to a set theorist as sets with structure, to a computer scientist as a collection of generalized states and transitions, to a cognitive scientist as a natural category (e.g., dog) consisting of a collection of instances and their relationships to each other, and so on. Such examples immediately suggest that a mathematical category consists of a collection of elements or objects and their relationships. Although this approach can provide immediate orientation toward the concept at hand, those aspects that are essential to the concept may be obscured by those aspects that are incidental. Indeed, with hindsight, much about the question of whether connectionist models implement classical symbol systems in their explanations or demonstrations of systematicity can be seen as a dispute between *models*, rather than *theories* of structure in the light of category theory. We will return to this point later in our assessment of the categorical approach.

An alternative approach is to start with the concept's definition from which concrete examples are instantiated. For mathematical concepts that have precise definitions, this (axiomatic) approach immediately identifies those properties that are necessary and sufficient for something to be an instance of that concept. However, applications may not be immediately obvious. Given that category theory may not be familiar to many cognitive scientists and the relative scarcity of applications to cognition, we steer an intermediate course with just enough formalism and examples to assess a categorical approach to the systematicity problem. Deeper and broader introductions to category theory can be found in many introductory books aimed at readers of various backgrounds, from those readers with more mathematical experience (Mac Lane, 1998; Awodey, 2010; Leinster, 2014) to those with less (Lawvere and Schanuel, 1997; Lawvere and Rosebrugh, 2003; Simmons, 2011), to those more comfortable with computational concepts (Arbib and Manes, 1975; Rydeheard and Burstall, 1988; Barr and Wells, 1990; Pierce, 1991; Walters, 1991; Crole, 1993), including functional programming (Bird and de Moor, 1997) and relational information systems (Spivak, 2014). There does not appear to be an introductory book for cognitive scientists, but see Ehresmann and Vanbremeersch (2007) Gomez-Ramirez (2014), and Magnan and Reyes (1995) for examples of general descriptions of cognitive systems in categorical terms. Since the central concept underlying a categorical explanation for systematicity is universal construction, and universal construction depends on the concepts of category and functor, we begin with the concept of a category, followed by functor, then universal construction.

### 2.1. Categories

Category theory starts with the concept of *category*. A category consists of a collection of entities, called *objects*, a collection of directed links between objects, called *arrows* (*morphisms* or *maps*), and a *composition operation*, denoted ○, that takes two “connected” arrows (where arrows are connected if the object at the end of one arrow is the object at the start of another arrow) and forms another arrow, subject to certain rules (axioms). Graphically, if there are objects and arrows 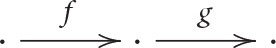
then there is a third arrow 
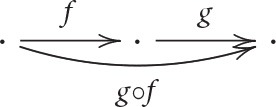
, i.e., the composition of *f* and *g*. Every object *A* is associated with an *identity arrow* from/to itself, denoted 1_*A*_. Composition (○) is required to be *associative* and so the identity arrows have the expected properties (see e.g., Awodey, 2010, p. 5): associativity simply means that the order of compositions does not affect the result, i.e., *f* ○(*g*○*h*) = (*f*○*g*)○*h*; and just like 0 as the identity for addition over the real numbers, ℝ, has the property that *x* + 0 = *x* = 0 + *x* for all *x* ∈ ℝ, the identity arrows have the analogous property that *f* ○ 1_*A*_ = *f* = 1_*B*_ ○ *f* for all *f* : *A* → *B* in a category.

This austere definition of a category appears to have little to do with cognition. Yet, cognitive processes are typically modeled as functions, or relations between sets of cognitive states, which can be treated in terms of particular kinds of categories. The category **Set** has sets for objects, functions for arrows, and function composition as the composition operation, i.e., (*g* ○*f*)*x* = *g*(*f*(*x*)). Cognitivists posit sets of symbolic or vector representations and functions that map between sets of representations as models of cognitive states and processes. Thus, a cognitive model can be constructed in **Set**. Comparing the size, or more generally the order of two entities is another common cognitive ability. A set *P* with a *partial order* (≤) defined over its elements *p* ∈ *P*, satisfying *p* ≤ *p* (reflexive), *p* ≤ *q* ∧ *q* ≤ *p* ⇒ *p* = *q* (antisymmetric), and *p* ≤ *q* ∧ *q* ≤ *r* ⇒ *p* ≤ *r* (transitive), is called a *poset* and written (*P*, ≤), and is also a category, as follows. The objects of a poset as a category are its elements *p*, and there is exactly one arrow *p* → *q* whenever *p* ≤ *q*. The identity arrows exist because a partial order is reflexive, and compositions of arrows can be defined because a partial order is transitive. The set of real numbers ℝ with the usual order (≤) constitutes a poset, hence a category, which is useful in the context of learning as optimization, as we shall see later. Some of our examples use a generalization of a poset, called a *preorder*, i.e., a set with a relation over elements that is reflexive and transitive (making a preorder a category), but not necessarily antisymmetric (Awodey, 2010, p. 9). For an introduction to categories using posets see Simmons (2011).

As we mentioned earlier, a formal definition can clarify the essential properties a category. For instance, counting considered as a succession of numbers, e.g., *succ*:2 → 3, is clearly a partially (totally) ordered set, and hence a category, but adding numbers considered as arrows, e.g., 2: * → * where addition is the composition operation, e.g., 2 ○ 3 = 2 + 3 = 5, without zero, i.e., 0: * → * is not a category, because there is no identity arrow. Often one can readily determine whether some collection of objects and relations is categorical from this relatively simple definition. Yet, the road from definition to cognitive application may not be so straight and narrow.

So, at this point, we remark on the relevance of the identity and associativity properties as a primer for a categorical approach to the systematicity problem. The requirement that every object be associated with an identity arrow seems trivial when these arrows don't appear to do anything. And, the requirement that multiple compositions are not sensitive to the order in which the operations are carried out does not seem particularly important when adding three numbers, such as (2 + 3) + 4 vs. 2 + (3 + 4). However, categorical identities afford equational reasoning among arrows just as 0 affords equational reasoning among arithmetic expressions, and associativity affords an alternative path to a solution that may otherwise be difficult to resolve. For instance, compare solving for *x* in the sequence of arithmetic equations

(1) $$\begin{array}{lll} 1+x & = & 2, \end{array}$$

(2) $$\begin{array}{lll} -1+\left(1+x\right) & = & -1+2, \end{array}$$

(3) $$\begin{array}{lll} \left(-1+1\right)+x & = & 1, \end{array}$$

(4) $$\begin{array}{lll} 0+x & = & 1, \end{array}$$

(5) $$\begin{array}{lll} x & = & 1 \end{array}$$

with solving for arrow *f*:*A* → *B* given *g*:*B* → *C* which is assumed to have the inverse *g*^−1^:*C* → *B*, i.e., $g^{-1}○g=1_{B}$, and *h*:*A* → *C* in the sequence of arrow equations

(6) $$\begin{array}{lll} g○f & = & h, \end{array}$$

(7) $$g^{-1}\;○(g\;○\;f)\;=g^{-1}\;○\;h,$$

(8) $$\begin{array}{lll} \left(g^{-1}○g\right)○f & = & g^{-1}○h, \end{array}$$

(9) $$\begin{array}{lll} 1_{B}○f & = & g^{-1}○h, \end{array}$$

(10) $$\begin{array}{lll} f & = & g^{-1}○h. \end{array}$$

Equations (3) and (5) are obtained from the associativity and identity properties for addition, respectively. Likewise, Equations (8) and (10) are obtained from the associativity and identity properties for composition. Thus, while associativity can seem obvious, it is critical to reasoning like that above, and it does not hold for every binary operation: e.g., *a* − (*b* − *c*) ≠ (*a* − *b*) − *c*. We will see next that these general properties are important to “grounding” and “preserving” relationships between elements across different hierarchical levels. This feature of category theory is what makes the categorical approach to systematicity quintessentially unique.

### 2.2. Functors

We introduced the concept of a category for computationalists as a collection of maps (functions) between sets of states. The concept of a state can be generalized to include cognitive processes. For example, a learning process can be considered as a map from one (generalized) cognitive state consisting of the currently available cognitive processes to another state including newly available cognitive processes. For instance, one can consider associative learning as a process that transforms one collection of associative capacities to another collection of associative capacities. In category theory, if a generalized state or object can be regarded as a category, then there are maps between such states or objects (categories), residing in a possibly larger category, whose arrows are called *functors*.

Functors are to categories as functions are to sets. A functor is a map *F* : **C** → **D** that sends the objects and arrows in **C** to (respectively) the objects and arrows in **D** so as to preserve identities and compositions: *F*(1_*A*_) = 1_*F*(*A*)_, identities; *F*(*f* ○ *g*) = *F*(*f*) ○ *F*(*g*), compositions. In this sense, functors are called “structure-preserving” maps that are “grounded” by the preservation of identities. A structure preserving map *f* between posets is a monotonic function, which preserves order, i.e., *p* ≤ *q* ⇒ *f*(*p*) ≤ *f*(*q*). Posets are categories, hence monotonic functions are functors. Note that any set *S* can be regarded as a *discrete category* whose objects are the elements *e* of *S*, and whose arrows are just the identity arrows 1_*e*_ : *e* → *e* for each *e* ∈ *S*. Then functions between sets become functors, whose action on objects is given by the function, and whose action on arrows is given by *f*(1_*e*_) = 1_*f*(*e*)_. The composition property follows trivially, since the only possible compositions are of the form 1_*e*_ ○ 1_*e*_ = 1_*e*_. However, not every (generalized) function from objects and arrows in one category to objects and arrows in another category is a functor, since not all such functions will preserve composition and identities.

That a function (between sets) can be considered as a functor may seem perplexing given that we introduced functors as a kind of higher-order map, i.e., a map between maps, which perhaps confusingly suggests that a map and a map between maps are the same thing when they are not. In category theory, a particular concept of one kind may appear as a different kind of concept in another category, e.g., a functor considered as an object in another category; and as we shall see, a universal construction considered as a special kind of object, in turn, considered as another kind of universal construction. What prevents category theory from descending into a death spiral of arbitrary abstraction is that every instance of a concept resides in a particular category. Apparent conceptual ambiguities are readily resolved by recalling the categorical context of the attending concept. Compare the categorical concept of an arrow as an object with the linguistic concept of a verb as a noun. We shall return to this point on context as it also plays an important role with regard to systematicity. The relationship between levels is also critical to the explanation, which we presage with the next two remarks.

First, the definition of a functor introduces another important consequence of identities, which is the “grounding” of one entity with respect to another. For instance, the natural numbers (ℕ) together with addition can be considered as a category that consists of a single object called * and one arrow *n*: * → * for each number *n* ∈ ℕ, including the identity arrow 0: * → *, and addition is the composition operation. Strings (“words”) of characters from an alphabet (*A*) together with concatenation (·) as composition can also be considered as a category that consists of one arrow *a*: * → * for each character *a* ∈ *A*, considered as a one-character word, a special symbol ϵ representing the empty string as the identity arrow ϵ: * → *, i.e., ϵ · *a* = *a* = *a* · ϵ, and all compositions, e.g., *b* ○ *a* is the two-character word, *ab*. In this situation, there is a *count* functor (here, denoted #) from the strings category to the numbers category that counts the number of characters in each string, e.g., #(*cat*) = 3. By preserving identity the number of characters in the empty word is meaningfully grounded at 0, i.e., #(ϵ) = 0, and compositionality is preserved as illustrated by the following example, #(ϵ · *cat*) = #(*cat*) = 3 = 0 + 3 = #(ϵ) + #(*cat*). Compare with the functor definition, *F*(*f* ○ *g*) = *F*(*f*) ○ *F*(*g*), where the first composition corresponds to concatenation and the second corresponds to addition.

Second, a functor preserves compositionality and so subsumes and further generalizes the forms of compositionality available in other theoretical approaches to cognition. Compositionality is a central feature of the classical approach to cognitive modeling, and compositionality can also be incorporated into connectionist models. The classical form typically employs some kind of juxtaposition, e.g., concatenation, of symbols representing constituent entities to form strings of symbols representing complex entities, so that the syntactic relations between symbols reflect the semantic relations between constituent entities. Analogously, the connectionist form employs an operation over vector spaces, e.g., tensor product (Smolensky, 1987), for combining vectors representing constituents to form vectors, matrices, or tensors representing complex entities. One can interpret a functor as a general means of constructing objects and arrows in one category from objects and arrows in another category that subsumes the kinds of grammatical constructions familiar to classical approaches, or vector-based constructions familiar to connectionist approaches. For instance, there is a functor that sends directed graphs as objects representing grammars in a category of such graphs to categories as objects, in a larger category, representing all possible strings generated by that grammar (Walters, 1991). A *tensor* is a (bi)functor (Mac Lane, 1998), cf. binary function, which generalizes the notion of tensor product used in connectionist models (see e.g., Smolensky, 1990; Halford et al., 1994). A functor can also be employed to combine both symbolic and subsymbolic forms of compositionality (Clark et al., 2008).

Although category theory affords more general notions of compositionality, the increased generality in and of itself is not what addresses the systematicity problem: *a fortiori*, if classical compositionality is too general to pin down just the systematically related capacities, then this categorical notion of (functorial) compositionality is also too general given that the latter subsumes the former. The power of the categorical approach stems not from increased generality, but from a unique combination of generality and specificity. To quote from one of its founders, “good general theory does not search for the maximum generality, but for the *right* generality” (Mac Lane, 1998, p. 108, emphasis ours). To paraphrase, we seek not sufficient conditions, but necessary and sufficient conditions for systematicity. This requirement leads naturally to the concept of *universal construction* (Mac Lane, 1998) as an explanation for systematicity (Phillips and Wilson, 2010), and we turn to this next.

### 2.3. Universal constructions

We said that systematicity is generally regarded as being derived from some ability to represent/process the common component of a collection of structurally related cognitive capacities. Category theory can model cognitive capacity as compositions of arrows in a category of some kind. Universal construction is the formal, category theory concept that captures the commonality among a collection of arrows and their compositions that are constructed from, or composed by a functor. Accordingly, universal construction is the basis of an explanation for systematicity.

Before presenting the general concept of a universal construction, we provide a basic intuition using a simple example of systematicity with respect to *ordered* pairs of shapes. In this example, if one has the capacity to represent the ordered pair of shapes (square, triangle), then one has the capacity to represent the ordered pair of shapes (triangle, square), assuming one has the capacity to represent the basic constituents square and triangle. Intuitively, each capacity is obtained from: (1) a common component that is associated with the pair schema (*, *), see next paragraph, (2) a component that is specific (unique) to each capacity, i.e., square as the first shape and triangle as the second shape in the (square, triangle) instance, and triangle as the first shape and square as the second shape in the (triangle, square) instance, and (3) the composition of components (1) and (2). As a universal construction, each capacity is obtained from: (i) an arrow that corresponds to the common pair schema, (ii) an instance-specific arrow obtained by a functorial construction from the arrows that correspond to the basic constituents square and triangle, and (iii) the composition of arrows (i) and (ii). The functorial constructions are obtained by a functor that constructs an arrow corresponding to square as the first shape and triangle as the second shape for the (square, triangle) instance, and an arrow corresponding to triangle as the first shape and square as the second shape for the (triangle, square) instance from the square and triangle arrows. Thus, systematicity derives from the fact that each capacity is indivisibly connected to the same arrow.

**Figure 1:**
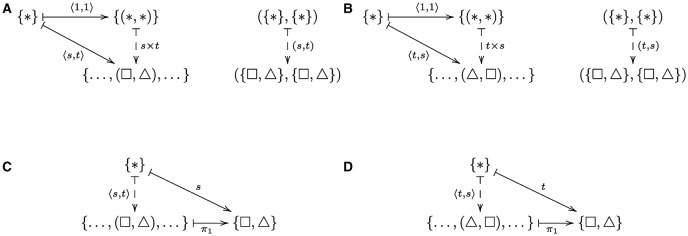
**Systematicity with regard to (A) the pair (square, triangle) and (B) the pair (triangle, square)**. The arrow 〈*s, t*〉 corresponds to the capacity to generate the representation for (square, triangle), indicated as the symbol pair (□, △). This arrow is composed of the arrow 〈1, 1〉, which is common to all shape pairs, and the arrow *s* × *t*. Arrow *s* × *t* is constructed by applying a functor (not shown) to the pair of arrows (*s, t*), which correspond to the basic capacity to generate a representation for square and triangle, respectively. The objects in these categories are sets and the arrows are functions, where ↦ indicates the action of the function on set elements, and dashed arrows indicate uniqueness, i.e., the only arrow that satisfies the equation 〈*s, t*〉 = *s* × *t* ○ 〈1, 1〉. The symbol * indicates an element whose name is unimportant. Note that {(*, *)} is a one-element set containing the (ordered) pair with the (unnamed) element * at both positions, and ({*}, {*}) is a pair with the one-element set {*} at both positions. Systematicity with regard to **(C)** the capacity to infer that the first shape of the pair (square, triangle) is square and **(D)** the capacity to infer that the first shape of the pair (triangle, square) is triangle, where the common arrow π_1_ is the projection that returns the first component of each pair. The arrows that are the sources of 〈*s, t*〉 and 〈*t, s*〉 in **(C,D)** are not shown.

The relationships between the various arrows are presented in the diagrams given in Figure 1, which also serves as in introduction to their use in category theory and subsequently in this paper. Figures 1A,B show the arrows corresponding to the instances (square, triangle) and (triangle, square), respectively. In a set-like category, i.e., a category whose objects are sets with possibly additional inter-element relations, an arrow (function) from a one-element set {*}, where * indicates that the identity of the element is unimportant, to a set *A* that is written $\bar{a}:*\mapsto a$ indicates a function that picks out, or points to, or generates the element *a* ∈ *A*. (The “mapsto” symbol ↦ indicates the action of a function of an element, i.e., *f* : *x* ↦ *y* indicates that *f*(*x*) = *y*, as exemplified in Figure 1.) Because there can be no ambiguity such arrows are simply denoted by the name of the element picked out. Hence, Figures 1A,B show that the capacities to represent the pairs of shapes (square, triangle) and (triangle, square), which correspond to arrows 〈*s, t*〉 and 〈*t, s*〉, are composed of a common arrow 〈1, 1〉 and the unique arrows *s* × *t* and *t* × *s*, respectively. Psychologically, one can interpret the 〈1, 1〉 arrow as the universal capacity to point to two objects in parallel, which one might associate with a notion of working memory capacity (see e.g., Cowan, 2001). The two unique arrows are constructed from a *product functor* (Mac Lane, 1998, p.38), denoted Π here, applied to the source arrows indicated as the right vertical arrows in the two diagrams. Conceptually, one can think of the product functor as providing an intrinsic role for the constituent arrows, *s* and *t*. The 〈1, 1〉 arrow identifies the universal role that each arrow plays in each pair of arrows in the current context. Note that it is not essential that the product functor be the conventional one that constructs the products *A* × *B* and *f* × *g* from the pairs (*A, B*) and (*f, g*), whence the universal arrow is a map to the object *A* × *B*; an alternative functor constructs products *B* × *A* and *g* × *f* from (*A, B*) and (*f, g*), whence the universal arrow is a map to *B* × *A*, because the general conditions for being a universal construction (discussed shortly) determine the universal arrow from the functor, as we shall see in the next section. Put another way, the functor contributes to the context in which the arrow is universal. Figures 1C,D show the universal arrow associated with a related instance of systematicity, the capacity to infer the first component of each pair. The universal arrow in this instance is a projection which returns the first component of each pair.

The diagrams also highlight another point which pertains to the implementation issue that has surrounded much of the systematicity debate: whether or not proposed alternatives to classical compositionality are implementations of the classical theory, or genuine alternative theories (see Smolensky, 1987, 1990; Fodor and Pylyshyn, 1988; Fodor and McLaughlin, 1990, for opposing sides of the debate). Note that in both instances of systematicity with regard to shape pairs, the category theory explanation is not committed to a particular symbolic or subsymbolic form of representation. One could replace the category **Set** with some other category that has products, like the familiar Cartesian product of sets *A* × *B*, such as the category of vector spaces, or topological spaces, or graphs (to name a few). A classicist may observe that the various compositional arrows, in fact, “token” their constituent arrows, which follows the classical form of compositionality: the tokening of constituents whenever their complex hosts are tokened. However, there is a crucial difference between categorical (universal) compositionality and classical compositionality, and that is the relationship between the arrows at different levels, viz. the universal arrow. Indeed, a universal arrow is formally defined as an arrow *from an object to a functor* (e.g., Figures 1A,B), or alternatively as an arrow *from a functor to an object* (e.g., Figures 1C,D) satisfying certain requirements. A universal arrow is also an object in another category (Mac Lane, 1998), which we explain and make use of in Section 4. The diagrams explicitly indicate the arrows within categories, i.e., the triangle of arrows which reside in one category and the stand-alone vertical arrow which resides in another category, and implicitly indicate the arrow (functor) between those categories. (NB. such diagrams usually show the objects and arrows in the form *A* → *B* to indicate the directed relationships between those objects, because objects need not be sets, nor have any internal components, as we saw in the case of posets, e.g., where the relationship 2 ≤ 3 constitutes the arrow 2 → 3. Implicit functorial relations are indicated by the application of the functor to the source objects, e.g., Π(*s, t*).) It is this inter-level relationship between arrows that is unique to the category theory approach, and warrants regarding this approach as an alternative theory, rather than an alternative implementation.

**Figure 2:**
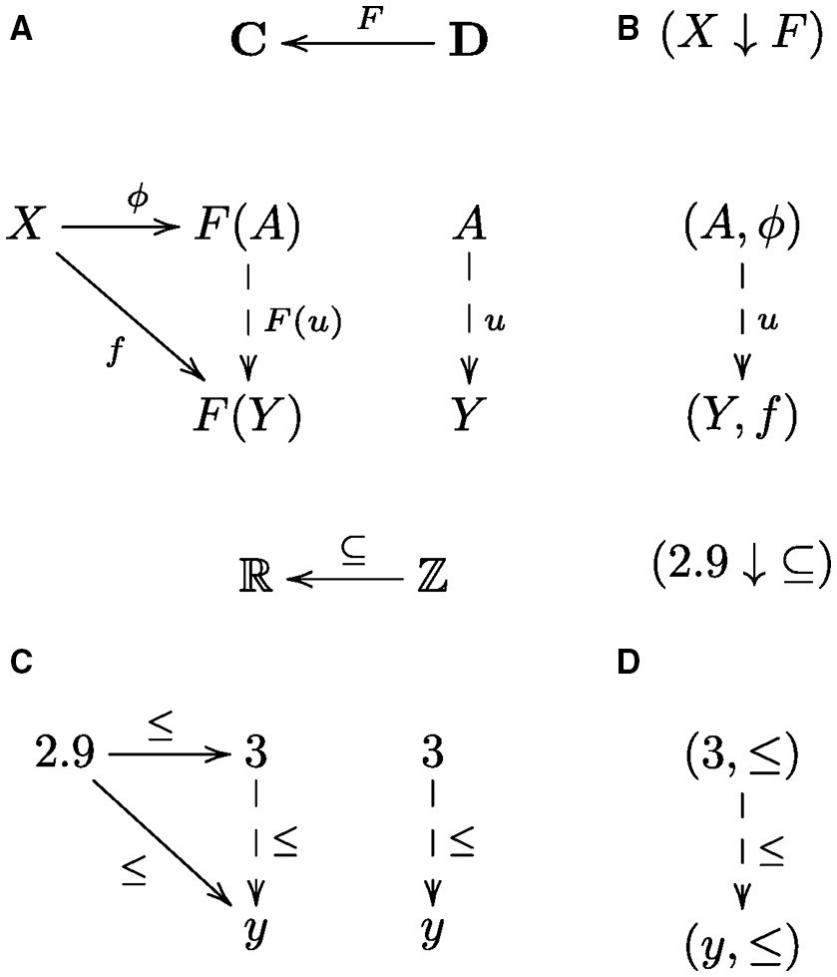
**Universal construction and comma category**. The diagram in **(A)** shows the general form of a universal arrow, which is the pair (*A*, ϕ), from an object *X* in a category **C** to a functor *F*: **D** → **C** from a category **D** to **C**. To be a universal arrow, as such, for every object *Y* in **D** and every arrow *f*: *X* → *F*(*Y*) in **C** there must exist a unique arrow *u*: *A* → *Y*, in **D**, such that *f* = *F*(*u*) ○ ϕ. The diagram in **(B)** shows the corresponding comma category, denoted (*X ↓ F*), whose objects are the pairs (*Y, f*) and arrows are the arrows *u* that uniquely satsify the “triangle” equation, *f* = *F*(*u*) ○ ϕ. The collection of objects in the comma category includes the universal arrow, (*A*, ϕ), because for *Y* set to *A* and *f* set to ϕ the triangle equation is uniquely satisfied by setting *u* to the identity arrow 1_*A*_. The universal arrow is the initial object in the comma category, which is straightforward to prove. Dashed lines indicate that the arrows are unique. An example universal construction **(C)** and corresponding comma category **(D)** is the universal arrow from the object (real number) 2.9 to the inclusion functor (function) from the integers to the real numbers (regarded as posets with the usual order ≤, hence categories) is the pair (3, ≤). The number 3 corresponds to the smallest integer greater than or equal to 2.9. In general, for a real number *x* ∈ ℝ, the object component of the universal arrow (*a*, ≤) from *x* to the inclusion function/functor is obtained by rounding up to the nearest integer *a* ∈ ℤ greater than or equal to *x*, i.e., obtained by the ceiling function, *a* = ⌈*x*⌉, and the corresponding comma category consists of all the integers *y* greater than or equal to *a*, i.e., the set {*y* ∈ ℤ|⌈*x*⌉ ≤ *y*}.

In the general situation, simply put, a universal arrow is an arrow ϕ from an object *X* to a functor *F* such that for every arrow *f* in a collection of arrows from *X* to *F* there is an arrow *F*(*u*) constructed from a unique arrow *u* by applying the functor *F* that composes with ϕ to give *f*, i.e., *f* = *F*(*u*) ○ ϕ (Mac Lane, 1998, p. 55). A diagram showing the general form and another example of a universal construction are given in Figure 2. An outline of a category theory explanation using this construction follows. The collection of *f*-arrows corresponds to a collection of systematically related cognitive capacities. The functor *F* constructs the constituent capacities *F*(*u*) that are composed with the universal arrow ϕ to realize each and every systematically related capacity *f* as the composition *F*(*u*) ○ ϕ. In other words, ϕ corresponds to the component that is common to all capacities *f*, and *F*(*u*) to the component that is specific to a particular *f*. The universal arrow is necessary in that by composing with this arrow we obtain *at least* all the arrows *f* in the collection of systematically related capacities; the universal arrow is sufficient in that we obtain *no more* than this collection. Moreover, this construction avoids the *ad hoc* aspects of classical and connectionist approaches, because there is no other choice of arrow that satisfies this condition, since *F*(*u*) is unique. In contrast, with classical and connectionist approaches there are (possibly many) other arrangements that *more or less* realize the targeted collection of systematically related capacities, whereby some (arbitrary) choice is needed to specify just that collection. Hence, universal arrows provide a category theoretical account of systematicity. Arrows in **Set** are functions; in posets/preorders they are orderings, but there are other categories with other kinds of arrows, e.g., arrows may be symbol transition rules, linear functions over vector spaces, graph transformations, or some other kind of process (or relationship). One can think of a universal construction, in the context of cognition, as an efficient (re)use of cognitive resources, since structurally related cognitive capacities all use the same component process ϕ, whether in symbolic, subsymbolic, or some other form.

From a classical perspective, categorical compositionality may appear as a (generalized) version of the classical *tokening* principle, hence a variation of classical compositionality, in that the instantiation of every compositional arrow (e.g., *g* ○ *f*) entails the instantiation of each of its constituent arrows (i.e., *f* and *g*); just as the instantiation of every complex symbol (e.g., “John loves Mary”) entails the instantiation of each its constituent symbols (“John,” “loves,” “Mary”). However, from a category theory perspective, not every composition involves a universal construction. That is, the composition of two arrows need not include a universal arrow. Universal construction provides the needed specificity that makes the categorical explanation a significant advance over classical and connectionist explanations vis-a-vis the unique existence property (Phillips and Wilson, 2010).

## 3. Universal construction, optimization, and learning

If systematicity derives from universal construction, then one may ask about the origins of universal constructions and hence origins of systematicity properties. Every universal construction can also be viewed as an optimization: conceptually, the “greatest common divisor” of a collection of arrows. And, indeed, conceptualizing universal construction as optimization provided the formal link between systematicity as a property of cognitive architecture (Fodor and Pylyshyn, 1988) and systematicity as a property of analogical reasoning, i.e., the preference for higher order relations as the basis for analogies (Gentner, 1983): both kinds of systematicity are derived from universal constructions (Phillips, 2014). In this section, we proceed by looking at universal constructions from the perspective of optimization, then optimization from the perspective of learning. This approach serves two purposes: (1) as a way of introducing the needed category theory concepts in the more familiar terms of learning as function approximation, and (2) to reveal what this explanation for systematicity means for theories of cognitive architecture.

### 3.1. Optimization as universal construction

**Figure 3:**
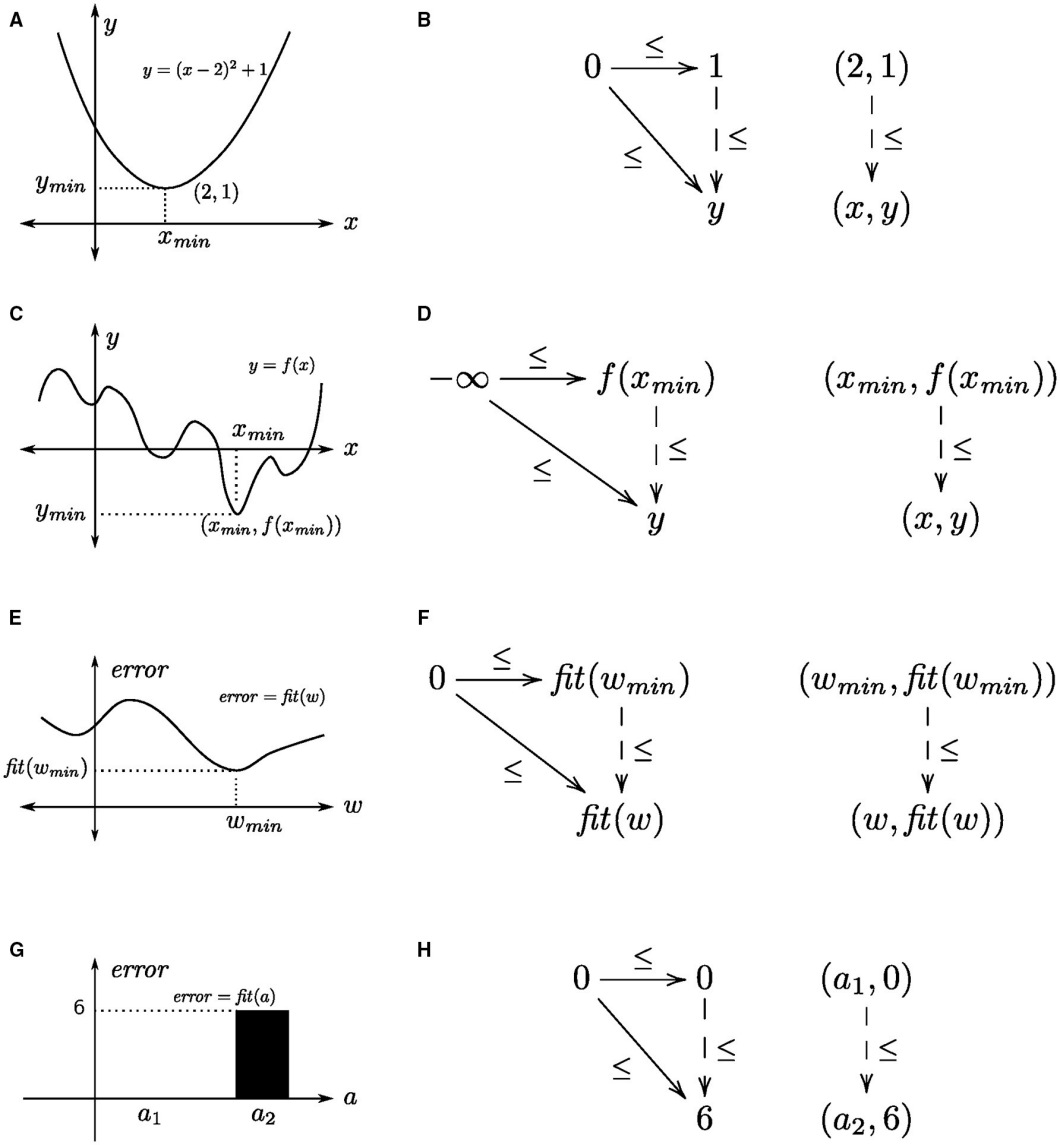
**Function minimization as a universal construction**. Within each row, the left panel shows the function and the right panel shows the corresponding universal construction. **(A,B)** a quadratic function, **(C,D)** an arbitrary function, **(E,F)** a fitness/error function over network connection weights, and **(G,H)** a generalized fitness/error function over a collection of algebras.

A straightforward way to understand the concept of a universal construction as an optimization is to consider the case of finding a global minimum (or, maximum) of a function. For concreteness, suppose we have the quadratic function $y=f_{Q}\left(x\right)={\left(x-2\right)}^{2}+1$, which has a global minimum at the point (*x, y*) = (2, 1). We will consider this function as a map from the set of real numbers, ℝ, to the set of non-negative real numbers, ℝ^+^, i.e., $f_{Q}:\mathbb{R}\to {\mathbb{R}}^{+}$. The *graph* of this function is the set of pairs $\Gamma \left(f_{Q}\right)=\left\{\left(x,f_{Q}\left(x\right)\right)|x\in \mathbb{R}\right\}\subseteq \mathbb{R}\times {\mathbb{R}}^{+}$, which contains, e.g., the points (0, 5) and (1, 2), and the minimum (2, 1). We can also define a partial order over this set of points as (*x, y*) ≤ (*x′, y′*) if and only if *y* ≤ *y′*, which makes Γ(*f*_*Q*_) a preorder, and therefore a category. The sets ℝ and ℝ^+^ are posets (so, categories), and so the map that returns the y-coordinate of each point in the graph is the monotonic function (so, functor) $\pi_{2}:\Gamma \left(f_{Q}\right)\to {\mathbb{R}}^{+};\left(x,y\right)\mapsto y$ (i.e., a *projection* onto the second coordinate), which preserves order. The minimum of *f*_*Q*_ is the object component of the universal arrow ((2, 1), ≤) from the object (point) 0 ∈ ℝ^+^ to the functor π_2_, see Figures 3A,B.

This categorical arrangement is quite general. It applies to any numerically valued function, which is straightforward to prove, and it need not be confined to continuous functions. In this case, we use the extended number line, ℝ^±∞^ = [−∞, ∞], i.e., the poset/category (ℝ, ≤) with the elements −∞ and ∞ adjoined. The minimum of a real-valued function *f*:ℝ^±∞^ → ℝ^±∞^, that is the point (*x*_*min*_, *f*(*x*_*min*_)), corresponds to the universal arrow from −∞ to π_2_, that is ((*x*_*min*_, *f*(*x*_*min*_)), ≤), see Figures 3C,D. We take the universal arrow to be from the point −∞ to ensure that we obtain the minimum of *f*. Uniqueness is given by the fact that as a preorder there cannot be more than one arrow from one object to another object in Γ(*f*); existence is given by the fact that ℝ^±∞^ is a total order, hence there must exist an arrow from the minimum to every point in Γ(*f*).

### 3.2. Learning as optimization

Having presented function minimization as a universal construction, the step to learning as function approximation, hence as a universal construction, is straightforward. Again, for concreteness, suppose we have a pair of input-target vectors *x* and *t* in real-valued vector spaces *X* and *T*, respectively, and *W* is the set of linear transformations (matrices) from *X* to *T*. In matrix notation, learning is finding a weight matrix *w* ∈ *W* that minimizes the squared length of the difference between the target and system's output (regarded as column vectors, i.e., one-column matrices) computed as the matrix product (·) of the weight and the input, i.e., the fitness function *fit*:*W* → ℝ^+^, where *fit*(*w*) = |*t* − *w* · *x*|^2^ for a particular input-target pair. More generally, the system's output for different weight matrices in *W* can be specified as a function *f*_*x*_: *W* → *T*, parameterized by input *x*, in which case the fitness function is $\text{fit}\left(w\right)=|t-f_{x}\left(w\right){|}^{2}$. An example and corresponding universal arrow is shown in Figures 3E,F. Hence, learning is finding the corresponding universal arrow, which we detail in the next section.

**Figure 4:**
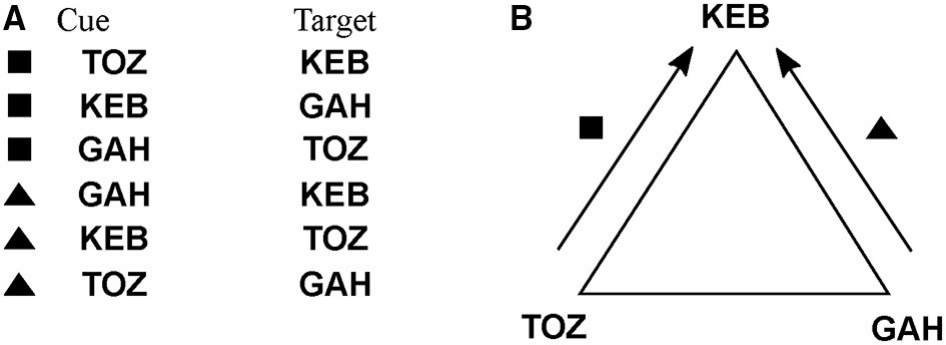
**An example cue-target prediction task for a schema induction paradigm showing (A) six cue-target pairs and (B) their geometric interpretation, where trigrams correspond to vertices of a triangle and shapes correspond to clockwise and anticlockwise rotation**.

A further generalization involves fitness functions whose domains are not numeric. This generalization also affords optimization (learning) over representations that have their own internal (algebraic) structure, e.g., group-like structures that have been employed in psychological tasks such as schema induction (Halford et al., 1998). A schema induction paradigm consists of a sequence of cue-target prediction tasks that conform to a common (algebraic) structure. An example is shown in Figure 4. Each task consists of six cue-target predictions, where each cue is a shape-trigram pair, and each target is a trigram, drawn from a set of two shapes and three trigrams, which are unique across tasks (Figure 4A). A geometric interpretation of the common structure across tasks is to regard each trigram as the vertex of a triangle, and each shape as a clockwise or anticlockwise rotation. For instance, the cue (■, TOZ) predicts the target KEB, which corresponds to a clockwise rotation that moves the bottom-left vertex to the top vertex. If we restrict to these shapes and trigrams, then there are two such algebraic structures, the one given in Figure 4 and the other one obtained by reversing the shape roles, which we denote as *a*_1_, *a*_2_ ∈ ***A***. The fitness function, *fit*: ***A*** → ℕ simply maps each algebraic structure to the number of mismatches between target, as specified by the task instance, and output, as specified by the given algebraic structure. Suppose the task instance is specified by *a*_1_, then *fit*(*a*_1_) = 0 and *fit*(*a*_2_) = 6, since there are six mismatches. The corresponding universal arrow is shown in Figures 3G,H.

## 4. A computational category theory approach to learning

We started with the question about the origins of universal constructions. Now that we have recast learning as a universal construction, we are in a position to present a novel treatment of learning, and hence the development of systematicity properties, as the process of finding the associated universal arrow. The computational principle that is presented is categorical in nature and motivated by the observation that every universal arrow can be viewed as a special object in a particular category. First, we explain this observation, which motivates the two algorithms that follow. The first algorithm is iterative, and pedagogical, serving as an easier to understand, concrete illustration of the computational principle. However, our interest is in the second algorithm, which is recursive, because this algorithm also derives from a universal construction via a category theory treatment of learning as (co)recursion.

**Figure 5:**
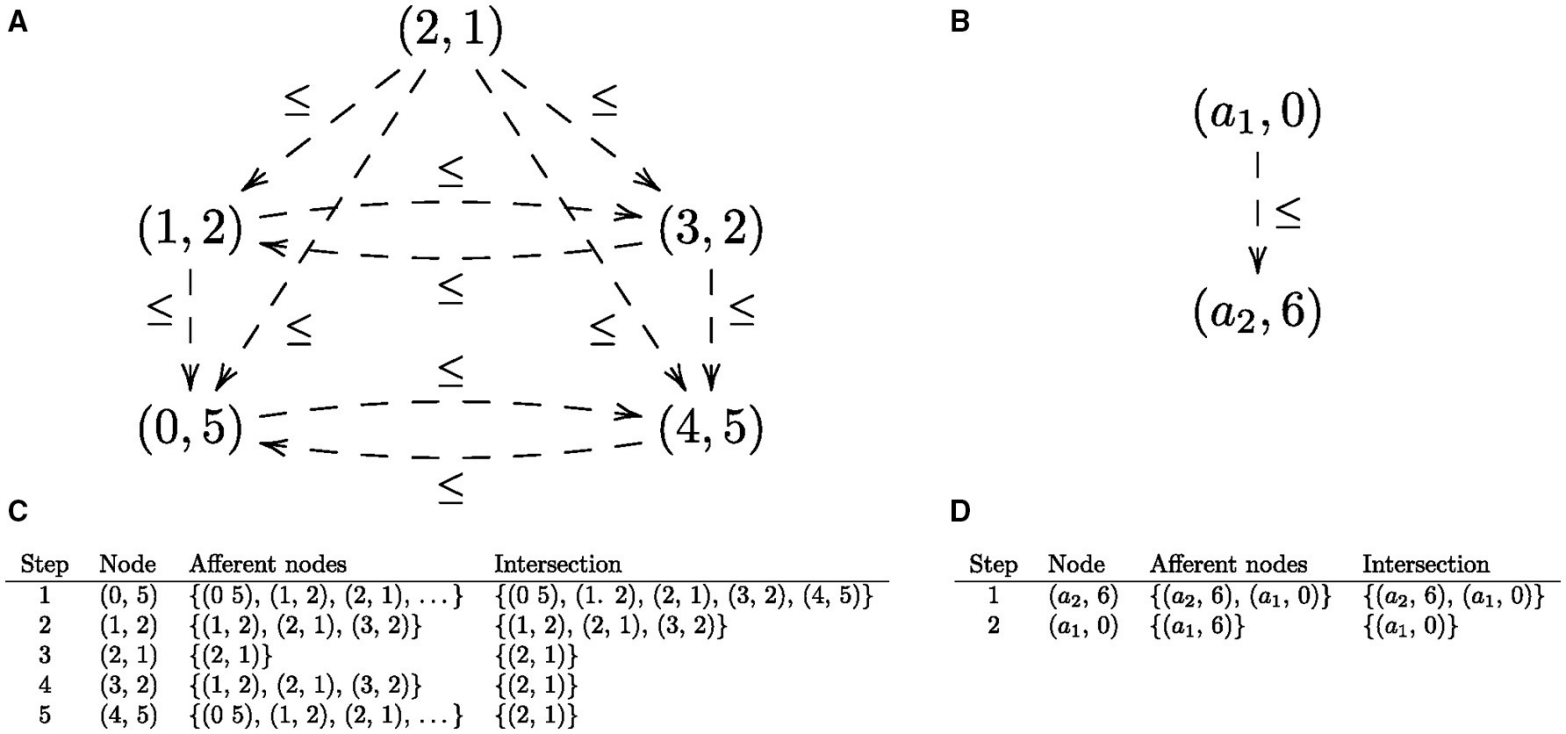
**(A)** The comma category corresponding to the universal construction shown in Figure 3B as a directed graph. **(B)** The comma category corresponding to the universal construction shown in Figure 3D as a directed graph. Edges corresponding to identity arrows are not shown. **(C,D)** The iterative process for finding the initial nodes in corresponding directed graphs.

We observe that every universal arrow corresponds to an *initial object* in the corresponding *comma category* (Mac Lane, 1998), see Figure 2. An initial object in a category (if it exists) is an object that has a unique arrow from it to every object in the category. For example, in a poset category such as the natural numbers with the usual order, (ℕ, ≤), the initial object is 0, because 0 ≤ *n* for every *n* ∈ ℕ. One can think of an initial object as a generalized “least element” in the sense that objects in a category are “ordered” by arrow direction: arrow *A* → *B* indicates that object *A* comes before object *B*, hence *A* is “less than” *B*, so an initial object is the least object in that category. Categories are closely related to graphs (in the sense of nodes and directed edges) via another kind of universal construction that is known as a pair of *adjoint functors* (Mac Lane, 1998): briefly, every directed graph can be extended to a category by adding an arrow for each connected path. Conversely, every category can be regarded as a directed graph by omitting (forgetting) the composition operation. The task of finding the universal arrow can be converted into the task of finding the object in the category (or, node in the corresponding graph) that has an arrow to every object in the category. Thus, finding a universal arrow is equivalent to finding a node in the corresponding comma category, viewed as a graph that has an edge to every node in the graph. For the universal arrows given in Figures 3A,D that is the top node in the corresponding graphs given in Figures 5A,B, respectively. In what follows, we'll assume that a graph has at least one node.

### 4.1. Iterative algorithm

The process of finding a node that is connected to every node in the given graph is straightforward for categories with finite numbers of objects and arrows: each graph node (vertex, *v*) is associated with the set of its afferent nodes, i.e., the set of nodes such that there is an edge from each node to *v*; and the intersection of all afferent node sets is the set of nodes such that each node has an edge to every graph node, i.e., a universal arrow. If the intersection is empty, then there is no universal arrow. The set of initial nodes is ${⋂}_{i=1}^{n}\text{afferent}\left(v_{i}\right)$, where *n* is the number of graph nodes, and *afferent*(*v*_*i*_) returns the set of afferent nodes of *v*_*i*_. Finding the initial nodes of a graph can proceed iteratively by initializing the variable that maintains the running intersection (*intersect*) to the set of graph nodes, and the index (*i*) to one. At each step, *intersect* is updated to be the intersection of itself and the set of afferent nodes for the current node, i.e., *intersect* ∩ *afferent*(*v*_*i*_). Examples are given in Figures 5C,D.

### 4.2. Recursive algorithm

Informally, the algorithm for finding initial nodes operates recursively on the graph's list of nodes. If this list is down to just one (head) node *h*, then the output is the set of afferent nodes for *h*, i.e., *afferent*(*h*); otherwise, if the list has a node *h* followed by a non-empty (tail) list *t*, then the output is the intersection of the afferent nodes of *h* with the output for the list of nodes *t*. For the example of finding the global minimum of the function $f_{Q}\left(x\right)={\left(x-2\right)}^{2}+1$, given in Figure 3A, whose associated comma category is shown in Figure 5C, the list of graph nodes is *l* = [(0, 5), (1, 2), (2, 1), (3, 2), (4, 5)]. The items in the list also constitute the elements of the set of afferent nodes for the head node (0, 5) of *l*. Hence, the set of initial nodes is the intersection of the afferent set for *h* and the set of initial nodes for the tail *t* of *l*, which is the list *t* = [(1, 2), (2, 1), (3, 2), (4, 5)]. Recursively applying this procedure yields the singleton set containing the initial node (1, 2), which is the minimum of the function *f*_*Q*_.

Since recursion has been given a systematic treatment in computational category theory (Manes and Arbib, 1986; Bird and de Moor, 1997), category theory can also be used to model recursion in cognition (Phillips and Wilson, 2012), so associative learning as recursion (Phillips and Wilson, 2015), as another kind of universal construction. The advantage of this category theory perspective on recursion is that we also begin to address another form of (second-order) systematicity: systematic capacity to *learn* cognitive capacities (Aizawa, 2003), which we have addressed elsewhere in the case of associative learning (Phillips and Wilson, 2015). A detailed review of a category theory approach to recursion is beyond the scope of this paper. Here, we just outline the main aspects used for our learning algorithm.

A category theory approach to recursion (Bird and de Moor, 1997) begins with an *endofunctor* on a suitable category, i.e., a functor from/to the same category. Functors can be viewed as constructors of objects/arrows in one category from the objects/arrows in another category; hence, endofunctors can be viewed as recursive constructors. For example, an endofunctor on the category **Set** can be used to build lists of elements taken from particular sets (e.g., lists of integers) and list-processing functions. Importantly, the collection of lists and list-based processes built from such endofunctors also forms a category, and this category has a universal construction. Very briefly, the objects in this category are *algebras* and the arrows are *algebra homomorphisms*. The universal construction in this situation is called an *initial algebra*, i.e., an initial object in this category of algebras and algebra homomorphisms. Because an initial algebra is an initial object, all list-based (recursive) processes are composed of this initial algebra, which affords systematicity of list-related cognitive abilities (Phillips and Wilson, 2012). That is, there is a unique algebra homomorphisms from the initial algebra to every algebra in the category. This unique algebra homomorphism is called a (list) *catamorphism*, or colloquially a *fold*.

A (non-empty) list *fold* is a higher-order function that takes two arguments and returns a function that “folds up” a given list into a value. The first argument is a unary function ϕ that is returned when the list contains a single item, and the second argument is a binary function ψ that is otherwise applied to the head and the fold of the rest of the list to return a value. That is,

(11) $$\begin{array}{l} fold(\phi ,\psi ):\{\begin{array}{ll} \left[v\right] & \mapsto \phi (v);\\ h\cdot t & \mapsto \psi (h,fold(\phi ,\psi )(t)). \end{array} \end{array}$$

where [*v*] indicates a list containing the one item *v*, and · constructs a list from an item and a list (e.g., 1 · [2, 3] = [1, 2, 3]). Here, ϕ is instantiated to the function *afferent*, and ψ to the function that computes the intersection of the set of afferent nodes of *h* and the set of initial nodes returned by the recursive call to *fold*. In Figure 5C, that is the intersection of {(0, 5), (1, 2), (2, 1), (3, 2), (4, 5)} and {(2, 1)}, which is the set {(2, 1)} containing the minimum of the function *f*_*Q*_. (Order is significant in a list, but not in a set.)

As was pointed out elsewhere (Phillips and Wilson, 2015), a catamorphic approach to learning as recursion makes an unrealistic assumption: the entire list of training data must be processed before any learning can take place, because *fold* is tail-recursive (i.e., the list is folded tail-first), so there is no sense of on-line learning. An alternative approach is to employ a closely related universal construction, called a *final coalgebra* (Rutten, 2000). All list-based *corecursive* processes are composed using this final coalgebra. That is, in the associated category, there is a unique *coalgebra homomorphism* from every coalgebra to the final coalgebra. This unique coalgebra homomorphism is called a (list) *anamorphism*, or *unfold*. The definitions of catamorphism and anamorphism are *dual*: one definition is obtained from the other by reversing the directions of the arrows. Again, the details are beyond the scope of this paper, however, this approach affords a systematic treatment of associative learning (Phillips and Wilson, 2015). It is straightforward to recast our algorithm for learning universal constructions into this anamorphic form, and thereby afford systematic on-line learning.

For reasons of space, we just state this corecursive version here. Further details on a category theory approach to corecursion (and recursion) for the purpose of modeling cognition can be found in Phillips and Wilson (2012), Text S1. A (non-empty) list *unfold* is a higher-order function that takes an argument and returns a function that “unfolds” a value into a list. The argument is a function *p*? → (ϕ, ψ) that applies predicate (boolean-valued function) *p*? to an input value *x* that: if successful, then terminates the unfolding process with the single-item list [ϕ(*x*)]; otherwise, if not successful, then continues the unfolding process by returning the list constructed from ϕ(*x*) and the output of *unfold* applied to the application of the function ψ to *x*. In symbols,

(12) $$\begin{array}{ll} unfold(p? & \to (\phi ,\psi )):\\ \;x & \mapsto \{\begin{array}{ll} \left[\phi (x)\right] & p?(x);\\ \phi (x)\;\cdot \;unfold(p?\to (\phi ,\psi ))(\psi (x)) & \text{otherwise}. \end{array} \end{array}$$

Here, the input *x* is a pair (*l, s*) consisting of the current list of graph nodes *l* and the current set of candidate initial objects, which is initially the set of afferent nodes of the first (head) node of *l*. The predicate *p*?(*x*) tests for a one-item list *l*. If *p*? returns true (i.e., *l* = [*h*]), then the function ϕ returns the pair consisting of the empty list and the intersection of *s* with the set of afferent nodes of *h*, i.e., ([], *s* ∩ *afferent*(*h*)), in which case *unfold*(*p*? → (ϕ, ψ)) returns the one-item list [([], *s* ∩ *afferent*(*h*))]. If *p*? returns false (i.e., *l* = *h* · *t*, where *t* is a non-empty list), then the function ψ returns the pair consisting of the list *t* and the intersection of the candidate initial object set *s* and the afferent nodes of *h*, i.e., (*t, s* ∩ *afferent*(*h*)), in which case *unfold* (*p*? → (ϕ, ψ)) returns the list ([], *s* ∩ *afferent*(*h*))·*unfold*(*p*? → (ϕ, ψ))(*t, s* ∩ *afferent*(*h*)). In effect, the first component of the pair (*l, s*) maintains the current graph node, and the second component maintains the current candidates for initial objects. Following the interpretation of anamorphisms as *generalized* state machines (Rutten, 2000), we interpret *unfold* as a learning process that unfolds over time, hence on-line learning.

## 5. Category theory implications and a new challenge

An advantage of a category theory approach is that it provides a unified explanation for systematicity across numeric and non-numeric (symbolic) cognitive domains. Thus, we can address questions of systematicity in other species without having to presuppose a capacity for language, thereby enabling other kinds of tests that would not be possible on humans. The implications of this universal constructions explanation depend on the nature of objects and arrows in the category modeling the domain of interest. In this section, we look at the implications of a category theory perspective for learning and systematicity, and a new challenge that it poses for cognitive science.

### 5.1. Systematicity and failure: (Empirical) cost/benefit implications

One potential benefit that is afforded to cognitive architectures with the systematicity property is greater cognitive capacity for the available cognitive resources. This advantage is accrued when structurally equivalent cognitive abilities are obtained without duplicating existing component cognitive processes. However, such increase in efficient use of cognitive resources depends on the relative costs of universal vs. non-universal constructions (Phillips, 2013). For instance, in a simple scenario where cognitive resources were calculated as a function of the arrows in a category, an advantage was afforded when the number of equivalent cognitive capacities exceeded two. That is a cost/benefit tradeoff. The cost is associated with the arrows that must be deployed to realize the target cognitive capacities; the benefit is stimulus-response predictability (Phillips, 2013). When the number of capacities (stimulus-response mappings) is small, a non-universal construction provides the same capacities at lower cost; when the number of capacities is large, a universal construction provides a better cost/benefit tradeoff.

This implication was tested with an experiment designed to modulate the cost/benefit tradeoff for a particular universal construction, which was a categorical *product* (Phillips et al., 2016). Participants were required to learn two series of cue-target prediction maps, where the cues were letter pairs, e.g., (*P, K*), and the targets were colored shapes, e.g., red-square. One series of cue-target maps conformed to a product map of the form *f* × *g*: *Char* × *Char* → *Color* × *Shape*, which is the product of maps *f*: *Char* → *Color* and *g*: *Char* → *Shape* (experiment condition). The other series conformed to a non-product map between the same sets of cues and targets, i.e., each map was not a product of a char-color map and a char-shape map (control condition). Cues and targets were unique across maps. Within each series, the number of cue-target pairs (i.e., map size) varied as the square of the number of elements in the stimulus sets: map size was *n*^2^ for *n* ∈ {3, 4, 5, 6}. The critical manipulation was map size order, which was either ascending or descending. For each map, about half of the mappings were used for training (i.e., feedback was given on the correct response) and about half for testing (no feedback). Performance on the novel (test) trials was used to evaluate whether participants induced the product construction. For the product condition, having learned the cue-target mappings in the training set, correct prediction was possible for all novel cue-target pairs in the testing set. Prediction was not logically possible in the non-product condition. The pertinent result was that in the product condition participants in the descending group showed significantly above chance-level prediction on the test trials for all map sizes, whereas participants in the ascending group only showed above chance-level prediction for the large map sizes, i.e., a failure of systematicity with regard to the smaller sized maps. This result is consistent with the categorical notion of a cost-benefit tradeoff: as the number of mappings to be learned increased, it became more advantageous to learn to construct the underlying product, which afforded having to learn fewer mappings to achieve greater predictability.

### 5.2. New challenge: Determining context

The existence of a universal arrow is a necessary and sufficient condition for having a collection of structurally related cognitive capacities modeled as arrows in some category; all such arrows factor through a universal arrow in an optimal way. However, this category theoretical explanation also depends on assuming suitable categories and a functor, i.e., the categorical context, with respect to which a construction is universal. A new challenge, then, is to explain the origin of this context. Here, we survey the nature of this challenge from a category theory perspective.

**Figure 6:**
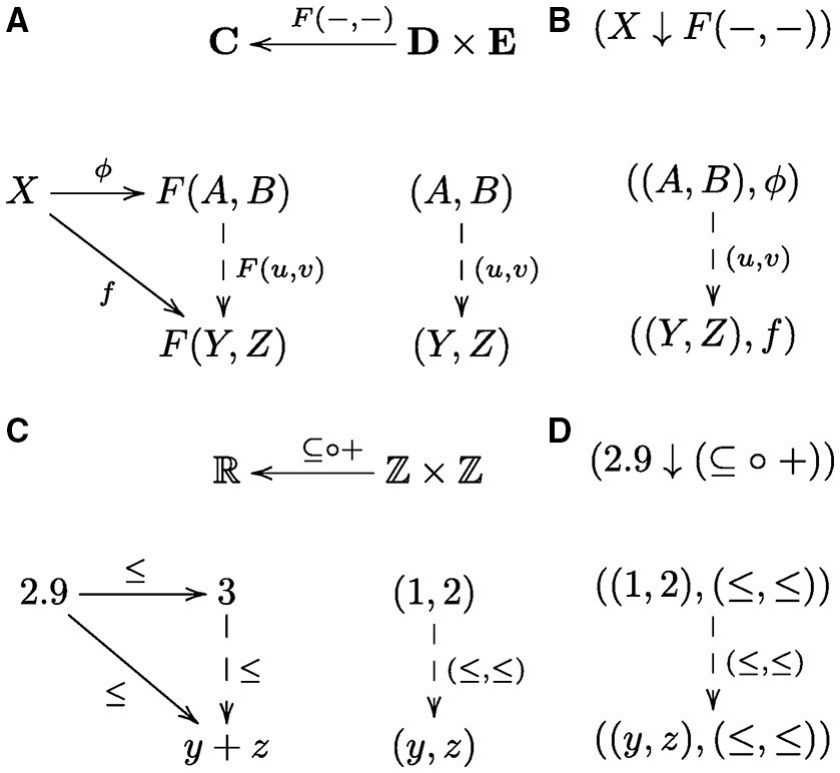
**Universal construction and comma category**. **(A)** Universal arrow from object *X* to bifunctor *F*(−, −) and **(B)** corresponding comma category (bifunctor). **(C)** The universal arrow from the object (point) 2.9 to the composition of the addition function/functor with the inclusion function/functor and **(D)** corresponding comma category.

As a framework, category theory affords a straightforward way to vary context: since functors compose, context can be expanded or restricted via composition of suitable functors. To expand context, for example, one can use a categorical analog of optimization with respect to two variables (binary function), which is a universal construction from an object to a *bifunctor*—bifunctor is to binary function as ordinary functor is to unary function. A bifunctor is a functor from a *product of categories* to a category. One can think of a product of categories **A** and **B** as an analog of a (Cartesian) product of sets in that the objects (and arrows) are all pairwise combinations of objects (and arrows) from **A** with those from **B**. The universal construction in this situation, and examples, are shown in Figure 6. To restrict context, for example, one can use an inclusion functor that picks out a suitable subcategory: inclusion functor is to subcategory as inclusion function is to subset. Note that a subcategory is a category, so not any subset of objects and arrows forms a subcategory, as the collection of arrows must be closed under composition, and therefore the choice is not arbitrary. An example of a universal arrow to the composition of two functors and corresponding comma category are given in Figures 6C,D. Restriction is important for learning and generalization (i.e., correct behavior on novel examples that were not available during learning). In the schema induction example, the category was restricted to just the two possible objects that conformed to the cyclic 3-group structure. In this case, only one training pair is needed to uniquely identity the target object (algebraic structure), and thus generalization is afforded in the form of correct target prediction for the other five cues. In the context of a more general category, e.g., the category of all groups, all training examples are needed uniquely identify the target, and thus no generalization is afforded.

For the theory, however, the new challenge then is to explain why context is restricted in the way that affords learning the needed universal construction. This challenge differs from the *strong systematicity* criterion (Hadley, 1994), which requires specifying a cognitive system, such as a neural network, that demonstrates correct behavior on novel examples with constituents that did not appear in the same (syntactic) position as in the training set. In the “John loves Mary” paradigm, for example, strong systematicity occurs if, after training on examples with John only in the lover position, success occurs on testing examples with John in the is-loved position. In contrast, the new challenge requires specifying the necessary and sufficient conditions that determine the context within which a categorical (recursive) learning architecture will necessarily acquire the needed universal construction.

In regard to this new challenge, one possible way forward is to develop the notion of costs and benefits more generally in a (categorical) framework. Cost is defined in terms of resources, which could include internal resources such as energy expenditure, and external resources such as number of training examples, or time. Benefit is defined in terms of the goals of the cognitive system. Such goals include, naturally, basic survival including predictability, but more esoterically, intellectual performance, which has long term survival value. Hence, in this framework, systematicity is determined by resource-constrained, goal-directed optimization. We further suggest that failures in systematicity are also the result of a perceived tradeoff. The phrasebook model of language learning as a counterexample to systematicity (Fodor and Pylyshyn, 1988) is a pertinent example. A tourist may opt to memorize a few key phrases to facilitate their travels, in lieu of expending the resources needed to learn a foreign language, because they perceive that the costs outweigh the benefits; a participant in a psychological experiment can willfully ignore task instructions, and so systematicity obtained via learning also depends on goals. We note, in closing, that every category theoretic construction belongs to a category of some kind. Thus, the category theory perspective presented here already suggests the form of an explanation for this new systematicity challenge. That is, a universal construction in a category of contexts. A natural approach toward addressing this challenge, then, is higher-order category theory (see e.g., Lambek and Scott, 1986) where categories and functors are (co)domains of other higher-order constructions and categories. One extension that may be particularly relevant to a notion of cost-benefit is *enriched category theory* (Kelly, 2005), where the relations between objects need not be a set of arrows, but a more general structure that nonetheless satisfies the rules for composition. For instance, as is well-known among category theorists, a *metric space* is an enriched category where the relation between each ordered pair of objects is a real number that indicates their “distance” (Lawvere, 2002). Thus, distance can be interpreted as the cost of proceeding from one object to the next.

## Author contributions

All authors listed, have made substantial, direct and intellectual contribution to the work, and approved it for publication.

## Funding

This work was supported by a Japanese Society for the Promotion of Science Grant-in-aid (26280051).

### Conflict of interest statement

The authors declare that the research was conducted in the absence of any commercial or financial relationships that could be construed as a potential conflict of interest.
